# Major Bleeding Events in Hospitalized COVID-19 Patients: A Retrospective Observational Study

**DOI:** 10.3390/medicina60050814

**Published:** 2024-05-15

**Authors:** Andrea Poloni, Giacomo Casalini, Giacomo Pozza, Andrea Giacomelli, Marta Colaneri, Giorgia Carrozzo, Beatrice Caloni, Cosmin Lucian Ciubotariu, Martina Zacheo, Andrea Rabbione, Margherita Pieruzzi, Federico Barone, Matteo Passerini, Anna Lisa Ridolfo, Giuliano Rizzardini, Andrea Gori, Spinello Antinori

**Affiliations:** 1 Department of Biomedical and Clinical Sciences, Università degli Studi di Milano, 20122 Milan, Italy; andrea.poloni@unimi.it (A.P.); giacomo.pozza@unimi.it (G.P.); giorgia.carrozzo@unimi.it (G.C.); beatrice.caloni@unimi.it (B.C.); cosmin.ciubotariu@unimi.it (C.L.C.); martina.zacheo@unimi.it (M.Z.); andrea.rabbione@unimi.it (A.R.); margherita.pieruzzi@unimi.it (M.P.); federico.barone@unimi.it (F.B.); andrea.gori@unimi.it (A.G.); spinello.antinori@unimi.it (S.A.); 2 III Infectious Diseases Unit, ASST Fatebenefratelli Sacco, Luigi Sacco Hospital, 20157 Milan, Italy; giacomo.casalini@asst-fbf-sacco.it (G.C.); annalisa.ridolfo@asst-fbf-sacco.it (A.L.R.); 3 II Infectious Diseases Unit, ASST Fatebenefratelli Sacco, Luigi Sacco Hospital, 20157 Milan, Italy; marta.colaneri@gmail.com (M.C.); matteo.passerini@unimi.it (M.P.); 4 I Division of Infectious Diseases, ASST Fatebenefratelli Sacco, Luigi Sacco Hospital, 20157 Milan, Italy; giuliano.rizzardini@asst-fbf-sacco.it; 5 Centre for Multidisciplinary Research in Health Science (MACH), Università degli Studi di Milano, 20122 Milan, Italy

**Keywords:** SARS-CoV-2, antiplatelet, age, anticoagulant, fatal bleeding

## Abstract

Thromboprophylaxis/anticoagulation treatment is often required in hospitalized COVID-19 patients. We aimed to estimate the prevalence of major bleeding events in hospitalized COVID-19 patients. This was a retrospective observational study including all COVID-19 hospitalized patients ≥18 years of age at one reference center in northern Italy. The crude prevalence (between February 2020–2022) of major bleeding events was estimated as the number of major bleeding episodes divided by patients at risk. Uni- and multivariable Cox models were built to assess factors potentially associated with major bleeding events. Twenty-nine (0.98%) out of 2,945 COVID-19 patients experienced a major bleeding event [prevalence of 0.55% (95%CI 0.37–0.79)], of which five were fatal. Patients who experienced a major bleeding event were older [78 years (72–84 IQR) vs. 67 years (55–78 IQR), *p*-value < 0.001] and more frequently exposed to anti-aggregating therapy (44.8% vs. 20.0%, *p*-value 0.002) when compared to those who did not. In the multivariable Cox model, age [per 1 year more AHR 1.05 (CI95% 1.02–1.09)] was independently associated with an increased risk of major bleeding events. A strict monitoring of older hospitalized COVID-19 patients is warranted due to the risk of major bleeding events.

## 1. Introduction

As of March 2024, the SARS-CoV-2 pandemic has caused nearly 7 million deaths worldwide, according to World Health Organization (WHO) [1]. When compared to other community acquired pneumonias [2,3,4], COVID-19 pneumonia exhibits a higher prevalence of venous thromboembolism, involving predominately the lungs, especially in the patients with severe disease [5,6,7]. The interplay between platelets, coagulation factors and immune system in response against systemic infections is known to be a host mechanism in which platelets are crucial in presenting the antigen and activating the host immune response, in particular neutrophils and monocytes [8]. The high incidence of thromboembolic events in SARS-CoV-2 infection seems to be linked to both host immune response and SARS-CoV-2 specific features, such as the interaction between the Spike protein and the ACE-2 receptor on the platelets’ surface and endothelial cells, which determine a higher risk of thromboembolic and cardiovascular events [9], a hypothesis which is also supported by autoptic findings [4,10]. 

Consequently, the most recent guidelines from WHO and the National Institutes of Health (NIH) recommend the implementation of thromboprophylaxis in hospitalized COVID-19 patients, preferably utilizing low-molecular-weight heparin (LMWH) to mitigate thromboembolic complications [11,12]. Nevertheless, major bleeding events have been reported as a consequence of thromboprophylaxis and anticoagulation in COVID-19 patients, especially when incremented or therapeutic dosages are prescribed [13,14,15], though the correlation between higher anticoagulant (AC) dose and incidence of major bleeding has not yet been fully depicted [16,17]. 

The evolution of the SARS-CoV-2 pandemic involved a reduction in mortality [18,19], with some authors reporting a lower incidence of thromboembolic events in second pandemic waves when compared to the first one [20,21] and a significant reduction in AC prescriptions in the omicron era [22]. 

The identification of COVID-19 patients at higher risk for major bleeding is required to optimize the clinical management of AC prophylaxis. Little is known about factors associated with major bleeding events in COVID-19 patients, while it is well known that there is a worse outcome of subjects experiencing a major bleeding complication during hospitalization, both in term of mortality and length of stay [23].

This study aims to estimate the period prevalence of major bleeding events in patients hospitalized for COVID-19, characterize the clinical outcomes of subjects experiencing this complication and identify potential factors associated with major bleeding events.

## 2. Materials and Methods

### 2.1. Study Design and Setting

We performed a monocentric retrospective observational study including all the patients hospitalized for SARS-CoV-2 infection at the infectious diseases department and at the intensive care unit (ICU) of Luigi Sacco Hospital (ASST-FBF-Sacco, Milan, Italy) from 20th February 2020 to 28th February 2022 (end of observation) enrolled in the Luigi Sacco COVID-19 registry [18,19,24,25,26]. 

### 2.2. Study Population

Inclusion criteria: (1) patients hospitalized at the study center; (2) patients with a positive molecular nasopharyngeal test for SARS-CoV-2 and COVID-19; (3) patients aged ≥ 18 years; (4) signing of informed consent for the inclusion of personal and clinical data in the database. Exclusion criteria: (1) patients hospitalized at the study center with a negative molecular nasopharyngeal sample for SARS-CoV-2; (2) patients aged < 18 years; (3) failure to sign the informed consent for the inclusion of personal and clinical data in the database. 

### 2.3. Definitions

For the present study, major bleeding was defined in accordance with Schulman et al. [27] as: (1) fatal bleeding and/or (2) symptomatic bleeding in a critical area or organ, such as intracranial, intraspinal, intraocular, retroperitoneal, intra-articular or pericardial, or intramuscular with compartment syndrome and/or (3) bleeding causing a fall in hemoglobin level of 2 g/dL or more, or leading to transfusion of two or more units of whole blood or red cells. Severity of COVID-19 disease at admission was defined according to the last updated NIH guidelines: mild, individuals who have any of the various signs and symptoms of COVID-19 (e.g., fever, cough, sore throat, malaise, headache, muscle pain, nausea, vomiting, diarrhea, loss of taste and smell) but do not have shortness of breath, dyspnea or abnormal chest imaging; moderate, individuals who show evidence of lower respiratory disease during clinical assessment or imaging and who have an oxygen saturation measured by pulse oximetry (SpO2) ≥ 94% on room air at sea level; severe, individuals who have SpO2 < 94% on room air at sea level, a ratio of arterial partial pressure of oxygen to fraction of inspired oxygen (PaO2/FiO2) < 300 mm Hg, a respiratory rate >30 breaths/min or lung infiltrates >50%; critical, individuals who have respiratory failure, septic shock and/or multiple organ dysfunction [12]. Thromboembolic event (TE) was defined as a positivity for a clot on a deep vein ultrasound or a positivity for thromboembolism of lungs or other body organs on computed tomography with contrast medium. Patients were classified into four waves according to the date of hospitalization: wave 1 from 21 February to 31 July 2020, wave 2 from 1 August 2020 to 31 January 2021, wave 3 from 1 February to 30 September 2021, wave 4 from 1 October 2021 to 28 February 2022 [28]. Comorbidities were derived from the previous clinical records of the patients, from the therapy that the patients underwent at hospital admission and from the new diagnosis that was possibly made during the hospital stay. In brief, we considered chronic obstructive pulmonary disease and restrictive lung disease as pulmonary comorbidities; heart disease was defined as a history of heart disorders such as heart failure, myocardial infarction or rhythm disorders; central nervous system diseases included comitial disorders, cognitive impairment, stroke (present or past with sequels), Alzheimer disease, medullary disease conditioning various grade of paralysis; diabetes was defined as hemoglobin A1c value ≥ 6.5% or plasma fasting glucose ≥ 126 mg/dL for two consecutive detections in the case of a new diagnosis; hepatic impairment was defined as a history of chronic liver disease (cirrhosis, autoimmune hepatitis, viral hepatitis); we considered immunosuppression in patients undergoing a chronic immunosuppressant or immunomodulant therapy, or in patients living with HIV having the CD4+ count below 200/mcl; cancer comorbidity was defined as a history of cancer disease still undergoing follow-up visits for risk of recrudescence or as an active cancer disease; chronic kidney disease (CKD) was defined as a history of eGFR < 60 mL/min/1.73 m^2^ for more than 3 months. Regarding AC management, our center did not have an internal protocol for AC dosages; the single physician decided the dosage of AC therapy considering the weight and the renal function of the single patient; for the present study, we considered the patient on AC prophylaxis dose or on AC treatment dose based on what was reported in the clinical records; when not explicated in the clinical records, we derived the type of AC therapy based on the dose of AC in relation to the weight and the renal function of patient. 

### 2.4. Data Collection

The characteristics of data collection applied to our study have been extensively described elsewhere [17,18,24,25,26]. Epidemiological, clinical, radiological and laboratory data about COVID-19 patients were prospectively recorded in an electronic database. Data about major bleeding events (AC or AP therapy at and during hospitalization, date and type of major bleeding, treatment of the major bleeding) were retrospectively retrieved for the purpose of the present study. 

### 2.5. Outcomes

The main outcomes of interest were to establish the risk factors related to major bleeding events and to evaluate how major bleedings effect major clinical outcomes, in particular length of stay, death and the maximum oxygen level needed by the patients during the hospital stay. 

### 2.6. Statistical Analysis

Continuous variables were described as median and 25th and 75th percentile, while categorical variables were described as frequency and percentage. The crude period prevalence of bleeding events was estimated as the number of major bleeding episodes divided by the patients at risk, with a corresponding 95% confidence interval (CI). Univariable Cox regression models were used to investigate the relationship between the age, biological sex, disease severity at hospital admission, antiplatelet and anticoagulant treatment at hospital admission and the main outcome of interest (major bleeding). Associations were estimated using hazard ratios (HRs) with their 95% confidence intervals (CIs). Multivariable Cox regression models were used to adjust the HRs for potential a priori chosen confounders, in accordance with the literature, including age, biological sex, disease severity at hospital admission, antiplatelet and anticoagulant treatment at hospital admission; the results were expressed as adjusted hazard ratios (AHRs) with their 95% CIs.

### 2.7. Ethical Statement

The study was approved by our ethics committee [Comitato Etico Interaziendale Area 1, Milan, Italy (Protocol No. 16088)].

## 3. Results

### 3.1. Characteristics of the Study Population

We included in the study 2945 patients, mostly males (62.3%) and European (85.5%), with a median age of 67 years (IQR 55–78). There were 554 (18.8%) hospitalized patients during the first wave, 839 (28.5%) during the second, 632 (21.5%) during the third and 920 (31.2%) during the fourth (Supplementary Figure S1). The median Charlson comorbidity index (CCI) was three (IQR 1–5): more than a half of the patients had a heart disease (55.1%), 15.6% had an underlying lung disease, 14.0% had diabetes mellitus and 9.3% had chronic kidney disease (CKD); mean BMI was 27.2 (SD 5.2) (Table 1). We classified the patients based on their severity of disease at the admission: mild (14.2%), moderate (39.7%), severe (27.5%) and critical (18.5%). Regarding the oxygen supplementation, 14.2% of the patients required mechanical ventilation, while 23.7% of them required a C-PAP or non-invasive ventilation (NIV) with high-flow nasal cannula (HFNC) (Table 2). A total of 458 patients (15.5%) were on AC therapy before the hospitalization, of which 58.9% were on AC treatment; regarding the indications, 212 (46.3%) were on AC treatment for atrial fibrillation, 155 (33.8%) were on AC therapy for COVID-19 diagnosis as outpatients (93.6% of them were on AC prophylaxis, while the remaining were on AC treatment), 40 (8.7%) were on AC therapy for TE prophylaxis and 31 (6.8%) for TE treatment, 11 (2.4%) were on AC treatment for prosthetic cardiac valve, 5 (1.1%) were on AC treatment for a cardiovascular event (three strokes, two acute coronary diseases) and finally, 4 (0.9%) were on AC treatment for thrombophilia. Most of our patients underwent an anticoagulant therapy during the hospitalization (85.4%): 70.9% of the patients received a prophylactic dose of AC and 14.5% a therapeutic dose of AC. LMWH was prescribed in 77.4% of the patients. Moreover, 596 patients (20.2%) were on antiplatelet (AP) therapy at admission, of which 38 (6.4%) were on double AP treatment for a recent cardiac acute disease, while the other 558 (93.6%) were on single AP therapy for primary or secondary prevention. Regarding vaccination, 4.4% of the patients had at least one dose, 10% had at least two doses and 2.5% had three doses.

### 3.2. Prevalence of Major Bleeding Events

We observed 29/2945 (0.99%) major bleeding events during the observation time, with an overall period prevalence of 0.55% (95% CI 0.37–0.79). The rate of bleeding events was 0.76% in patients undergoing the prophylactic dose and 2.34% in the treatment dose. The prevalence of major bleeding events was 1.46% in the critically ill patient’s subgroup (no patients underwent extracorporeal membrane oxygenation). 

We compared the baseline demographic and clinical-epidemiological features between patients who developed and did not develop a major bleeding event during the hospitalization. We found that older age [median 78 years (72–84 IQR) vs. 67 years (55–78 IQR); *p* < 0.0001], higher Charlson comorbidity index [median 5 (3–7 IQR) vs. 3 (1–5 IQR); *p* < 0.0001], chronic kidney disease (24.1% vs. 9.2%; *p* = 0.015), heart disease (86.2% vs. 54.8%; *p* = 0.001) and AP therapy (44.8% vs. 20.0%; *p* = 0.002) were more frequent in those who experienced a major bleeding event when compared to those who did not. We did not see a statistically significant difference in the risk of major bleedings regarding sex (*p* = 0.865), ethnicity (*p* = 0.669), severity of disease (*p* = 0.612), waves (*p* = 0.464) or AC therapy before the admission to our center (*p* = 0.072). We found a correlation, though barely significant, between a lower BMI and the risk of major bleeding (26.7 vs. 27.2, *p* = 0.0399).

### 3.3. Characterization of Subjects with a Major Bleeding Event

Regarding the 29 patients who developed a major bleeding event, eight (27.6%) were on AC therapy before the admission to our hospital. Seven patients were on AC treatment for atrial fibrillation, and one was on AC prophylaxis for reasons related to COVID-19 infection (this patient received the diagnosis with a nasal–pharyngeal sample as an outpatient and started an AC prophylaxis with LMWH before the hospitalization). Thirteen patients (44.8%) were on AP treatment at admission. At admission, 26 (89.6%) patients started or already were on AC therapy: 16 patients (55.6%) were on AC prophylaxis with LMWH, nine (34.6%) were on AC treatment with LMWH and one patient was on oral AC treatment with warfarin. Only one patient (3.4%) who experienced a major bleeding event did not start an AC therapy after the admission (the patient was asymptomatic for COVID-19 disease). During the course of hospitalization, 17 patients (58.6%) introduced the AC treatment dose: 16 patients were already on AC prophylaxis and they increased the AC dosage until the treatment dose was reached; one patient was not on AC prophylaxis and started the AC treatment dosage directly. Regarding these patients, six increased the dose for a documented pulmonary TE, five for an increase in the d-dimer level, three for new evidence of atrial-fibrillation, two for clinical worsening and one for documented deep-vein thrombosis (inferior limbs). The median time between admission and bleeding event was 16 days (IQR 8–23). We calculated the median time of bleeding from admission in patients who were on AP therapy at admission (11 days, IQR 7–16) and were not (16 days, IQR 11–24), without a statistically significant difference (*p* = 0.18). Twenty-six (89.7%) patients were undergoing an AC treatment at the time of the bleeding event. The more frequent site of hemorrhage was at a deep muscular level (58.6%), followed by gastro-intestinal bleeding (20.6%), brain and retroperitoneal bleeding (6.9%, respectively) and lungs and bladder bleeding (3.4%, respectively). Eleven patients (37.9%) underwent embolization through interventional radiology; a fatal bleeding occurred in five patients (17.2%). 

### 3.4. Outcome of Subjects with a Major Bleeding Event

We found that length of stay (median days) was significantly longer in patients with a major bleeding event [35 (IQR 27–49) vs. 14 (IQR 8–24), *p* < 0.0001)] (Table 3). Death was more frequently observed in patients with major bleeding (34.5% vs. 18.0%, *p* = 0.039). On the contrary, we did not find significantly differences regarding maximum oxygenation needed during the hospitalization between the two groups. 

### 3.5. Cox Models of Factors Associated with Major Bleeding Events

We performed a Cox regression model to assess factors associated with major bleeding events in our cohort study (Table 4). In the univariate analysis, we found age [per 1 year more HR 1.05 (CI95% 1.02–1.09)], AP therapy at admission [HR 2.65 (95%CI 1.27–5.52)] and chronic kidney disease [HR 2.71 (95%CI 1.15–6.36)] were significantly associated with the development of a major bleeding event. In the multivariable model, after adjusting for an a priori defined set of confounders and potential mediators, we found that age was independently associated with an increased risk of major bleeding events [per 1 year more AHR 1.05 (95%CI 1.02–1.09)]. AC treatment at hospital admission showed a non-significant higher adjusted hazard ratio of major bleeding events [AHR 1.18 (95% CI 0.24–5.89)], when compared to no AC exposure; moreover, also AP therapy at admission showed a non-significant higher hazard ratio of major bleeding events [AHR 1.98 (95% CI 0.92–4.30)]. Severity of disease was not associated with an increased major bleeding risk both in univariate and in multivariate analysis [moderate vs. mild HR 1.16 (0.37–3.66), AHR 1.73 (0.53–5.71); severe vs. mild HR 0.71 (0.20–2.53), AHR 1.12 (0.30–4.15); critical vs. mild HR 1.13 (0.34–3.77), AHR 2.51 (0.68–9.31)].

**Table 3:** Outcome comparison between patients who developed and who did not develop a major bleeding event.

	No Major Bleeding Events n = 2916	Major Bleeding Event n = 29	Total n = 2945	*p*-Value
**Length of Stay (day), median (IQR)**	14 (8–24]	35 (27–49)	14 (8–24)	<0.001
**Death, n (%)**	524 (18.0)	10 (34.5)	534 (18.1)	0.039
**Maximum oxygen supplementation, n (%)**				
Nasal cannula	558 (19.9)	6 (23.1)	564 (20.0)	0.649
Venturi mask	567 (20.2)	6 (23.1)	573 (20.3)	
Reservoir mask	196 (7.0)	1 (3.8)	197 (7.0)	
C-PAP or NIV (HFNC)	661 (23.6)	8 (30.8)	669 (23.7)	
Mechanical ventilation	398 (14.2)	4 (15.4)	402 (14.2)	

Abbreviations: n = number, SD = standard deviation, IOT = NIV = non-invasive ventilation. HFNC = high-flow nasal cannula.

**Table 4:** Multivariable Cox regression model of factors associated to major bleeding events.

	HRs (95% CIs)	AHRs (95% CIs)
**Male (vs. female)**	1.07 (0.50–2.32)	1.24 (0.56–2.76)
**Age (per one year more)**	1.05 (1.02–1.09)	1.05 (1.02–1.09)
**Prophylactic anticoagulant at the admission (vs. no)**	0.76 (0.22–2.63)	0.49 (0.10–2.36)
**Therapeutic anticoagulant at the admission (vs. no)**	1.94 (0.56–7.10)	1.18 (0.24–5.89)
**Antiplatelet therapy (yes vs. no)**	2.65 (1.27–5.52)	1.98 (0.92–4.30)
**Chronic kidney disease (yes vs. no)**	2.71 (1.15–6.36)	1.78 (0.74–4.34)
Moderate disease * (vs. mild)	1.16 (0.37–3.66)	1.73 (0.53–5.71)
Severe disease * (vs. mild)	0.71 (0.20–2.53)	1.12 (0.30–4.15)
Critical disease * (vs. mild)	1.13 (0.34–3.77)	2.51 (0.68–9.31)
**Wave ^+^ 2 (vs. wave 1)**	0.95 (0.28–3.25)	0.82 (0.19–3.57)
**Wave 3 (vs. wave 1)**	1.04 (0.28–3.87)	1.15 (0.24–5.50)
**Wave 4 (vs. wave 1)**	1.91 (0.62–5.86)	1.87 (0.44–8.01)

Abbreviations: CKD = chronic kidney disease. * In accordance with [12]. ^+^ In accordance with [28].

## 4. Discussion

AC prophylaxis is recommended in COVID-19 pneumonia [12]. However, major bleeding events have been reported, especially in critically ill COVID-19 patients requiring therapeutic AC dosages [13]. In our cohort, we observed 29 major bleeding events, with a prevalence of 0.99% in the overall cohort and 1.46% in the critically ill patient subgroup; we observed a prevalence of bleeding events of 0.76% in patients undergoing AC prophylactic dose and 2.34% in patients undergoing a treatment dose; the overall period prevalence of major bleeding was 0.55%. Our prevalence of major bleeding events is lower than the one reported in the literature. In particular, the prevalence of major bleeding was reported as 3% in an overall cohort of Danish patients hospitalized for COVID-19 infection, increasing to 11% in the ICU patient subgroup [29]. Other retrospective studies also showed a higher prevalence of major bleeding events when compared with our cohort, as reported by H. Al-Samkari et al. (2.3% overall and 5.6% in ICU patients) [30], M. Lucijanic et al. (3.2% overall and 7.6% in ICU patients) [31], P. Lamouche-Wilquin et al. (8% in ICU patients) [16], R. Halaby et al. (14.3% in ICU patients) [13] and L. Russell et al. (6% in ICU patients) [32]. Moreover, clinical trials have also reported higher rates of major bleedings while evaluating the efficacy of different AC doses, as reported by Lawler et al. [33] and Lopes et al. [34]. In a meta-analysis by D. Jimenez evaluating the bleeding risk in hospitalized patients with COVID-19, the pooled incidence of major bleeding was reported as 3.9% [35].

The observed that the lower prevalence of major bleeding events in our study can be attributed to the lower proportion of patients receiving high doses of AC treatment compared to the existing literature. In our cohort, overall, only 14.5% of the patients received a higher than prophylactic dose of AC therapy, compared to the 46.7% in the Danish cohort [29] and the 34.1% in the M. Lucijanic cohort [31]. Considering only ICU hospitalizations, 71.3% of the patients in R. Halabay et al. [13] underwent a treatment AC dose, while 76% of ICU patients in L. Russell’s cohort received a higher than standard prophylactic dose of AC therapy [32]. In our cohort, only 53.1% of severe COVID-19 patients received treatment exceeding prophylactic AC doses. Our findings align with a meta-analysis by J. Ena and V. Valls [36], where the rate of major bleeding events was reported as 0.99% in not critically ill patients undergoing prophylactic dosages of anticoagulant therapy. Also, another meta-analysis by S. Zuily et al. [37], comparing fixed and weight-based dose anticoagulant prophylaxis, found a low rate of major bleeding (1% in the weight base group and 1.3% in the fixed dose group), comparable with our findings. 

At our center, we routinely prescribed standard prophylactic AC dose for patients hospitalized for COVID-19, except in patients hospitalized in ICU, where a higher AC dose was usually applied. Given the substantial number of critically ill COVID-19 patients, especially during the initial and subsequent waves, many were managed in the infectious disease ward, receiving the standard prophylactic AC dose at our center. This could explain the lower proportion of critically ill patients undergoing AC treatment in our cohort. Our decision to consistently employ the standard prophylactic AC dose for non-ICU hospitalized patients was influenced by evidence indicating no mortality benefit with an AC treatment dose as a prophylaxis compared to the standard dose, particularly in non-critically ill patients, despite an elevated risk of major bleeding events [38,39,40]. 

Major bleeding complications were observed more frequently in patients undergoing the treatment dose AC (87.9% of the patients with a bleeding event were on AC treatment at the time of the bleeding); this finding, although not statistically significant (AHR 1.18, CI 0.24–5.89) probably due to the overall low number of major bleeding events, is in accordance with the literature [14,32,34,41], highlighting the known necessity of awareness for major bleeding complications especially in patients undergoing AC treatment. 

We interpreted the statistical significance (*p* = 0.0399) of lower BMI as a risk factor for major bleeding as having little clinical relevance, although a statistical significance was found. In our opinion, lower BMI in patients who experienced a major bleeding event is related to the older age and the greater CCI in this subgroup, both factors contributing to a loss in body mass and a lowering in weight, and both factors associated with bleeding risk in our analysis [median age in bleeding event subgroup 78 years (72–84 IQR) vs. 67 years (55–78 IQR), *p* < 0.0001; median CCI in bleeding event subgroup 5 (3–7 IQR) vs. 3 (1–5 IQR); *p* < 0.0001].

Age was significantly associated with major bleedings in our cohort [HR 1.05 (CI 1.02–1.09); AHR 1.05 (CI 1.02–1.09)]. The risk for major bleeding in this category of patients was incremented by 0.05% more per each year. Although, with the result barely significant, we thought that the clinical relevance of the finding is consistent with the higher risk of bleeding in older patients receiving an AC drug that has been well established in the literature [42]. The increased major bleeding risk in older patients depends both on comorbid medical conditions (such as congestive heart failure, cerebrovascular disease, hepatic and renal disease, diabetes mellitus, history of bleeding and anemia) rising the risk of major bleeding [43], and on the aging process itself [44]. Our data reinforce the need for careful monitoring in older patients with COVID-19 disease when starting an AC drug.

CKD was found to be associated with major bleeding events in the univariate analysis (HR 2.71, CI 1.15–6.36), though we did not confirm this finding in the multivariate analysis (AH 1.78, CI 0.74–4.34), probably because the overall low number of bleeding events. Moreover, we did not stratify patients on the CKD severity due to the low numbers. It is known that patients suffering from CKD have an increased risk of bleeding events [45] and the role of AC drugs in rising this risk in patients with severe CKD (eGFR < 30 mL/min) is described in a meta-analysis by W. Lim et al. [46], explaining this finding with the lower renal clearance and the consequent accumulation of anti-Xa heparin in severe CKD patients.

We found a two-fold higher risk for major bleeding in patients undergoing AP treatment at the time of hospitalization in the univariate analysis [HR 2.65 (95%CI 1.27–5.52)], but we did not confirm this data in the multivariate analysis, although there was the non-significant estimated two-fold higher risk [AHR 1.98 (95% CI 0.92–4.30)] when compared to individuals not exposed to AP at the time of hospital admission. This finding is in accordance with what is reported in the literature, both for critically ill [47] and not critically ill patients with COVID-19 infection [48], and, also, in patients not hospitalized for COVID-19 disease [42,49]. It is known that the AP effect of the more common AP drugs have a time-to-platelet function recovery after drug interruption of about 5–7 days [50], making the summatory effect on bleeding risk of AC therapy and chronic AP therapy at admission nearly inevitable. Regarding this, we found that patients with an AP therapy at admission experienced the major bleeding event earlier than patients without it, though without a statistical significance, probably due to the low numbers of major bleeding events [11 days (IQR 7–16) vs. 16 days (IQR 11–24), *p* = 0.18)]. Our results bring to attention the need for evaluation, patient by patient, of the possibility of interrupting AP therapy for the period in which the patient is undergoing AC therapy (both at standard and anticoagulant dose), especially in AP therapy for primary prevention, and the need to consider patients who interrupt AP drugs during an AC therapy potentially being at higher risk for early bleeding events. 

Deaths and length of hospital stay both increased in patients with a major bleeding event when compared with patients without it. The poor outcomes of patients undergoing a major bleeding event is reported for COVID-19 patients by Nakamura et al. in the sub-analysis of the CLOT-COVID study [23]; moreover, in-hospital major bleeding is a known predictor of in-hospital mortality also in other clinical settings [51,52]. Our data reinforce the importance of a careful patient selection before the inception of AC treatment, considering the risk of a major bleeding complication with its implication in terms of worse clinical outcomes.

### Limitations

Our study has a number of limitations. (1) As mentioned above, we extrapolated the data about the major bleeding events retrospectively, with the risk of events missing and a possible underestimation. (2) In our center, we did not have an internal protocol for the LMWH dose, leading to a wide heterogeneity in LMWH prescriptions, especially for the treatment dose; moreover, we did not have an internal protocol about anticoagulation therapy in patients with elevated D-dimer or suspected pulmonary TE when the execution of contrast medium CT was not able to be performed. (3) We did not gather the data about the TE for all the cohort because the data would underestimate the real number of TE due to the difficulty in the execution of the contrast medium CT in severe and critically ill patients for most of the study period due to logistical reasons. (4) Some patients incremented the AC therapy after the finding of elevated D-dimer levels: we did not report the data about D-dimer levels in the study cohort because we did not have the parameter for all the patients. (5) We did not calculate the bleeding risk in relation to the severity of CKD.

## 5. Conclusions

Major bleeding events in hospitalized patients with COVID-19 increased the length of stay and the risk of death. Older age is associated with a significant risk for major bleeding events. Also, patients on AP therapy at admission and with CKD hospitalized for COVID-19 disease must be carefully monitored for major bleeding while prescribed with AC drugs. A careful monitoring for signs and symptoms of major bleeding and a careful evaluation of bleeding risk are needed in these subgroups of patients to minimize major bleeding events and the unfavorable outcome that these complications may determine for the patients.

## Figures and Tables

**Table 1:** Epidemiological, clinical and treatment features of patients hospitalized for SARS-CoV-2 infection.

	Overall n = 2945	No Major Bleeding Events n = 2916	Major Bleeding Event n = 29	*p* Value (<0.05)
**Male, n (%)**	1834 (62.3)	1.815 (62.2)	19 (65.5)	0.865
**Age, median (IQR)**	67 (55–78)	67 (55–78)	78 (72–84)	<0.001
**BMI, mean (SD)**	27.2 (5.2)	27.2 (5.2)	26.7 (5.7)	0.0399
**Country of birth, n (%)**				
Europe	2517 (85.5)	2491 (85.5)	26 (89.7)	
Asia	159 (5.4)	158 (5.4)	1 (3.4)	
Africa	114 (3.9)	114 (3.9)	0 (0.0)	
South America	154 (5.2)	154 (5.2)	2 (6.9)	0.669
**Comorbidities, n (%)**				
Pulmonary	458 (15.6)	454 (15.6)	4 (13.8)	0.996
Central nervous system	68 (13.1)	68 (13.2)	0 (0.0)	0.974
Cardiovascular	1623 (55.1)	1598 (54.8)	25 (86.2)	0.001
Diabetes mellitus	412 (14.0)	406 (13.9)	6 (20.7)	0.437
Hepatic	107 (3.6)	105 (3.6)	2 (6.9)	0.656
Immunosuppression	210 (7.1)	210 (7.2)	0 (0.0)	0.255
Neoplasia	371 (12.6)	364 (12.5)	7 (2.41)	0.109
Chronic kidney disease	275 (9.3)	268 (9.2)	7 (24.1)	0.015
**Charlson comorbidity index, median (IQR)**	3 (1–5)	3 (1–5)	5 (3–7)	<0.001
**Wave, n (%) ^+^**				
1	554 (18.8)	550 (18.9)	4 (13.8)	
2	839 (28.5)	832 (28.5)	7 (24.1)	
3	632 (21.5)	627 (21.5)	5 (17.2)	
4	920 (31.2)	907 (31.1)	13 (44.8)	0.464
Disease severity at admission *, n (%)				
Mild	420 (14.2)	416 (14.3)	4 (13.8)	
Moderate	1170 (39.7)	1159 (39.7)	11 (37.9)	
Severe	809 (27.5)	803 (27.5)	6 (20.7)	
Critical	546 (18.5)	538 (18.4)	8 (27.6)	0.612
**COVID-19 vaccination doses, n (%)**				
One dose	130 (4.4)	129 (4.5)	1 (3.4)	
Two doses	293 (10.0)	289 (10.0)	4 (13.8)	
Three doses	72 (2.5)	70 (2.4)	2 (6.9)	0.389
**Anticoagulant therapy at admission, n (%)**	458 (15.5)	450 (15.4)	8 (27.6)	0.072
**Antiplatelet therapy at admission, n (%)**	596 (20.2)	583 (20.0)	13 (44.8)	0.002

List of abbreviations: n = number, IQR = interquartile range, BMI = body mass index, SD = standard deviation, CNS = central nervous system, CKD = chronic kidney disease. ^+^ In accordance with [28]. * In accordance with [12].

**Table 2:** Features of patients who experienced a major bleeding event.

Overall	n = 29
**AC treatment before hospital admission, n (%)**	8 (27.6)
**AP treatment before the admission, n (%)**	13 (44.8)
**AC treatment at admission, n (%)**	28 (96.5)
**Type of AC treatment at admission, n (%)**	
Coumadin	1 (3.8)
Enoxaparin prophylactic dose	16 (55.6)
Enoxaparin therapeutic dose	9 (34.6)
**Increased AC treatment during the hospitalization, n (%)**	17 (58.6)
**Hospitalization in ICU setting, n (%)**	2 (6.9)
**Time between admission and bleeding event, day (IQR)**	16 (8–23)
**Site of bleeding, n (%)**	
Deep muscular	17 (58.6)
Retroperitoneal	2 (6.9)
Gastrointestinal	6 (20.7)
Bladder	1 (3.4)
Brain	2 (6.9)
Lungs	1 (3.4)
**Therapeutic dosage of AC at the time of bleeding event, n (%)**	26 (89.7)
**Bleeding event needing embolization, n (%)**	11 (37.9)
**Fatal bleeding, n (%)**	5 (17.2)

Abbreviations: n = number, AC = anticoagulation, AP = antiplatelet ICU = intensive care unit, IQR = interquartile range.

## Data Availability

Data will be shared upon reasonable request.

## Supplementary Materials

The following supporting information can be downloaded at: https://www.mdpi.com/article/10.3390/medicina60050814/s1, Supplementary Figure S1. Flow chart of patients inclusion and data management.
