# Supplementary figures and images for: Major Bleeding Events in Hospitalized COVID-19 Patients: A Retrospective Observational Study
Source: Medicina (Kaunas). 2024 May 15;60(5):814. doi: 10.3390/medicina60050814 (PMC11122796; doi:10.3390/medicina60050814)

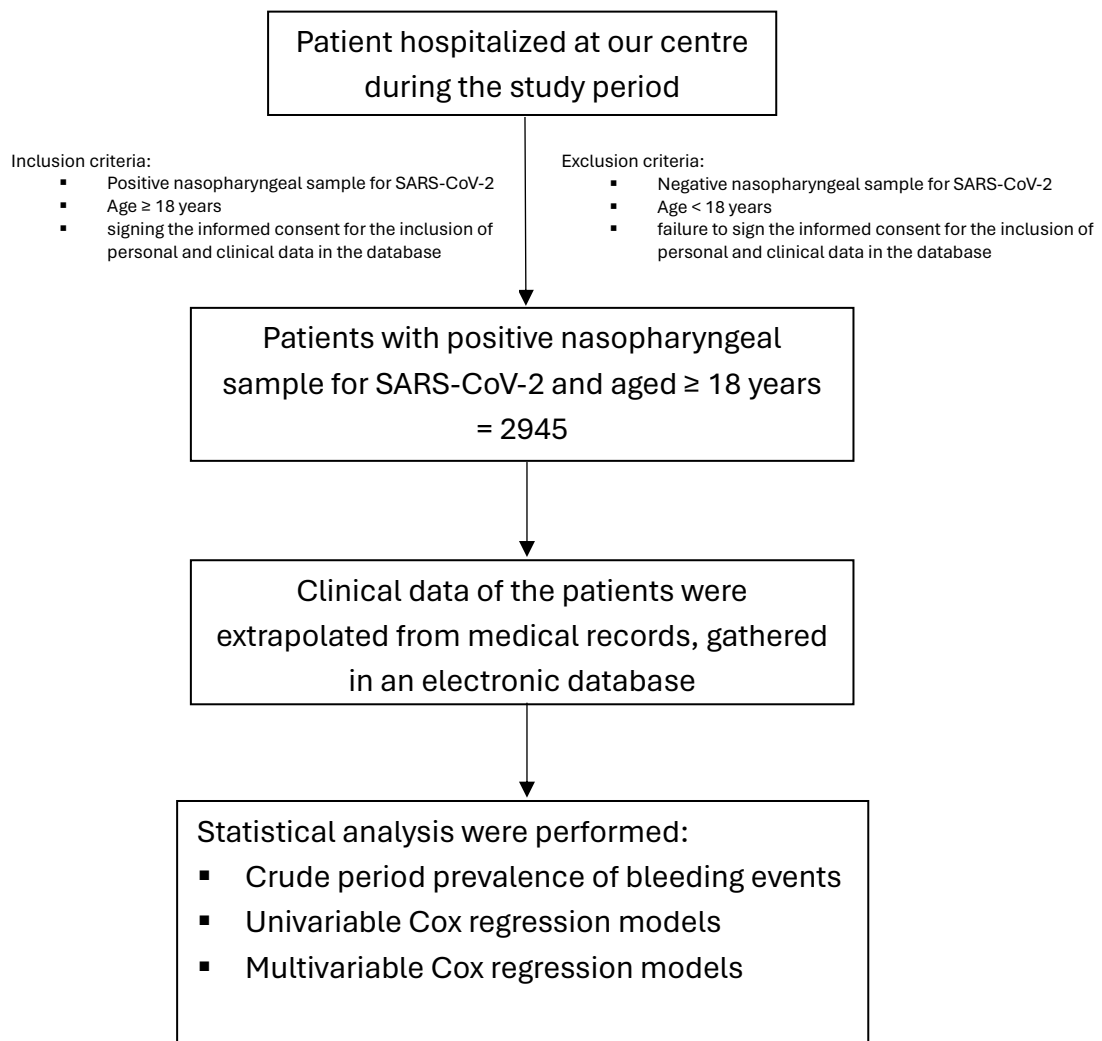

**Supplementary Figure S1.** Flow chart of patients inclusion and data management.

Supplement: Supplementary file 1 [file medicina-60-00814-s001.zip › medicina-2992199-supplementary.pdf]
